# The effects of goal-directed fluid therapy based on dynamic parameters on post-surgical outcome: a meta-analysis of randomized controlled trials

**DOI:** 10.1186/s13054-014-0584-z

**Published:** 2014-10-28

**Authors:** Jan Benes, Mariateresa Giglio, Nicola Brienza, Frederic Michard

**Affiliations:** Department of Anaesthesia and Intensive Care Medicine, The Faculty of Medicine and University hospital in Plzen, Charles University Prague, Alej Svobody 80, 306 40 Plzen, Czech Republic; Department of Emergency and Organ Transplantation, Anaesthesia and Intensive Care Unit, University of Bari, Policlinico, Piazza G. Cesare 11, 70124 Bari, Italy; Critical Care, Edwards Lifesciences, 1 Edwards Way, Irvine, CA USA

## Abstract

**Introduction:**

Dynamic predictors of fluid responsiveness, namely systolic pressure variation, pulse pressure variation, stroke volume variation and pleth variability index have been shown to be useful to identify in advance patients who will respond to a fluid load by a significant increase in stroke volume and cardiac output. As a result, they are increasingly used to guide fluid therapy. Several randomized controlled trials have tested the ability of goal-directed fluid therapy (GDFT) based on dynamic parameters (GDFTdyn) to improve post-surgical outcome. These studies have yielded conflicting results. Therefore, we performed this meta-analysis to investigate whether the use of GDFTdyn is associated with a decrease in post-surgical morbidity.

**Methods:**

A systematic literature review, using MEDLINE, EMBASE, and The Cochrane Library databases through September 2013 was conducted. Data synthesis was obtained by using odds ratio (OR) and weighted mean difference (WMD) with 95% confidence interval (CI) by random-effects model.

**Results:**

In total, 14 studies met the inclusion criteria (961 participants). Post-operative morbidity was reduced by GDFTdyn (OR 0.51; CI 0.34 to 0.75; *P* <0.001). This effect was related to a significant reduction in infectious (OR 0.45; CI 0.27 to 0.74; *P* = 0.002), cardiovascular (OR 0.55; CI 0.36 to 0.82; *P* = 0.004) and abdominal (OR 0.56; CI 0.37 to 0.86; *P* = 0.008) complications. It was associated with a significant decrease in ICU length of stay (WMD −0.75 days; CI −1.37 to −0.12; *P* = 0.02).

**Conclusions:**

In surgical patients, we found that GDFTdyn decreased post-surgical morbidity and ICU length of stay. Because of the heterogeneity of studies analyzed, large prospective clinical trials would be useful to confirm our findings.

**Electronic supplementary material:**

The online version of this article (doi:10.1186/s13054-014-0584-z) contains supplementary material, which is available to authorized users.

## Introduction

It has been known for a while that mechanical ventilation may induce cyclic changes in left ventricular stroke volume and arterial pressure [1]. Many experimental and clinical studies have demonstrated that the magnitude of the arterial pressure waveform variation is highly dependent on blood volume [2]. The idea to use the systolic pressure variation (SPV) or the pulse pressure variation (PPV) not only to track changes in blood volume, but also to predict fluid responsiveness (defined as the hemodynamic response to a fluid load), emerged 15 years ago [3-5]. Tavernier *et al*. [5] demonstrated that SPV is a better predictor of fluid responsiveness than left ventricular end-diastolic area, an echocardiographic marker of cardiac preload. Michard *et al*. [3] demonstrated that PPV is an accurate predictor of fluid responsiveness, dramatically better than cardiac filling pressures, and slightly but significantly better than SPV. Since then, many clinical studies have confirmed the value of SPV and PPV to predict fluid responsiveness [2]. Others dynamic parameters, mainly the stroke volume variation (SVV) measured by pulse contour methods [6] and the pleth variability index (PVI) derived from pulse oximetry [7], have also been proposed and used with success to predict fluid responsiveness. Today, most hemodynamic monitors calculate automatically and display these dynamic parameters. According to published peer-reviewed surveys, their clinical use to guide fluid therapy increased from 1% in 1998 [8] to 45% in 2012 [9].

If the ability of dynamic parameters to predict fluid responsiveness is now hardly disputable (pending the limitations to their use being respected), whether their use is associated with improved quality of care and outcome remains an open question. In 2007, Lopes *et al*. [10] were the first to demonstrate that intra-operative goal-directed fluid therapy (GDFT) based on PPV monitoring is able to decrease post-operative complications and hospital length of stay in patients undergoing major abdominal surgery. However, the following year, Buettner *et al*. [11] failed to reproduce these results, although their study design was very similar. Since then, several other randomized controlled trials (RCTs) have been performed where fluid therapy was driven by the use of dynamic parameters. These studies have yielded conflicting results. Therefore, we performed the present meta-analysis in order to clarify if GDFT based on dynamic parameters (GDFTdyn) may decrease post-surgical morbidity when compared to standard fluid management.

## Material and methods

### Eligibility criteria

The study was performed in adherence to the PRISMA statement (for the full PRISMA statement checklist see the supplemental digital content in Additional file 1) [12], no ethics board approval was deemed necessary for a meta-analysis of previously published studies. Eligible studies were searched according to the following criteria:

#### Type of participants

Adult (age 18 years or over) patients undergoing surgery were considered. Studies involving mixed population of critically ill or non-surgical patients were excluded.

#### Type of intervention

Intervention was defined as GDFT based on dynamic parameters (GDFTdyn).

#### Type of comparison

RCTs comparing the effects of GDFTdyn and standard fluid management were considered.

#### Type of outcome measures

The primary outcome measure was post-surgical morbidity. Morbidity was defined as the proportion of patients with one or more post-surgical complication. The specific post-surgical infectious, cardiac, respiratory, renal and abdominal complications, as well as ICU and hospital length of stay were assessed as secondary outcome variables. Abdominal complications included both gastrointestinal and liver complications.

#### Types of studies

No language, publication date, or publication status restrictions were imposed.

### Information sources

Different search strategies were performed to retrieve relevant studies by using MEDLINE, The Cochrane Library and EMBASE databases (last update September 2013). No date restriction was applied for MEDLINE and The Cochrane Library databases, while the search was limited to 2006 to 2013 for EMBASE database [13]. Additional trials were searched in The Cochrane Library and in the DARE databases and the reference lists of previously published reviews and retrieved articles.

### Search

We used the following search terms to search all trials: randomized controlled trial, controlled clinical trial, goal directed, goal oriented, goal target, cardiac output, cardiac index, oxygen delivery, oxygen consumption, cardiac volume, stroke volume, fluid therapy, fluid loading, fluid administration, optimization, pulse pressure variation, pleth variability index, stroke volume variation, systolic pressure variation. The search strategies used for MEDLINE, The Cochrane Library and EMBASE databases are shown in the supplemental digital content in Additional file 2.

### Study selection

Two investigators (MTG, NB) examined at first each title and abstract to identify potentially relevant articles. The eligibility of the retrieved full-text articles was independently determined by three investigators (JB, NB, FM). Only trials where dynamic predictors were used to titrate fluid administration were considered for analysis. All identified articles were in English so no text translations were necessary.

### Data collection process

Data were independently collected by two investigators (JB, MTG) with any discrepancy resolved by re-inspection of the original article. To avoid transcription errors, the data were input into statistical software and rechecked by a third investigator (NB).

### Data items

Data abstraction included number of patients, type of surgery, dynamic parameter used to guide fluid therapy, technology used to measure dynamic parameters, type of fluid administered, as well as the outcome variables described above.

### Risk of bias in individual studies

The Scottish Intercollegiate Guidelines Network (SIGN) checklist for RCTs was used to evaluate the methodological quality of RCTs [14]. The SIGN checklist was independently filled by two investigators (MTG, JB) and whenever different, the study was further assessed in order to reach consensus. A double plus (++) denotes studies very unlikely to have bias, a single plus (+) denotes studies where bias is unlikely, and a minus (−) studies with high risk of bias. A double plus was assigned to studies that adequately described all the criteria of randomization, concealment, blinding, intention-to-treat analysis, and predefined outcomes, whereas a single plus was given to studies meeting only four out of the five criteria. The adequacy of these five criteria is strongly associated with bias reduction [15,16]. With regard to blinding, studies in which the outcome variables were collected by investigators not aware of the intra-operative fluid strategy were considered adequately masked [17].

### Summary measures and planned method of analysis

Meta-analytic techniques (analysis software RevMan, version 5.2 Cochrane Collaboration, Oxford, England, UK) were used to combine studies using odds ratios (OR) and 95% confidence intervals (CI) for dichotomous variables, and weighted mean difference (WMD) and 95% CI for continuous variables. A statistical difference between groups was considered to occur if the pooled 95% CI did not include one for the OR. An OR less than one favored GDFTdyn when compared with standard fluid treatment. Two-sided *P* values were calculated. A random-effects model was chosen for all analyses. Statistical heterogeneity and inconsistency were assessed by using the Q and I^2^ tests, respectively [18,19]. When the *P* value of the Q-test was <0.10 and/or the I^2^ was >40% heterogeneity and inconsistency were considered significant [20].

## Results

### Study selection

**Figure 1:**
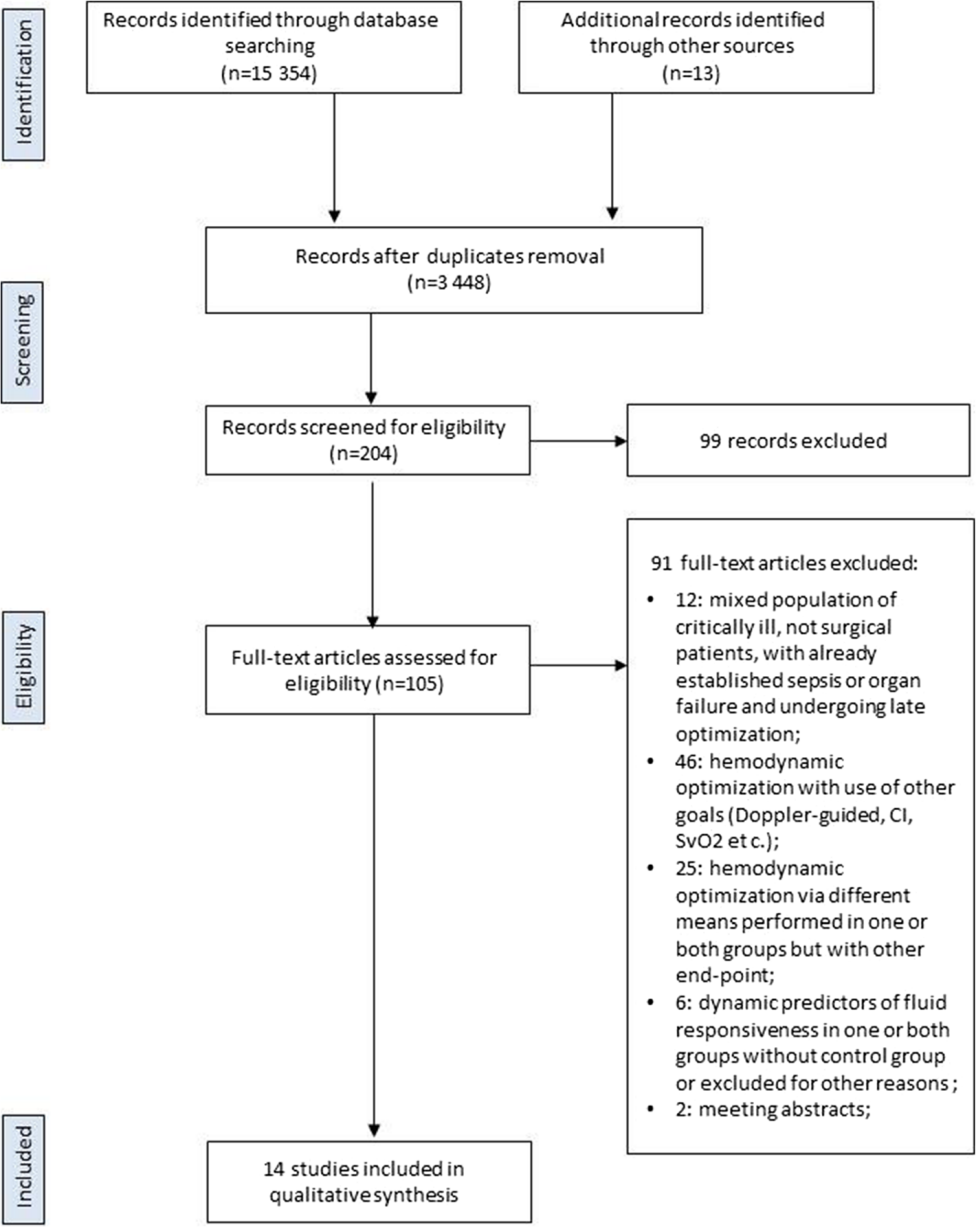
**Flow diagram illustrating search strategy.** CI: cardiac index; SvO_2_: central venous saturation; SVV: stroke volume variation.

The search strategies identified 3,297 (MEDLINE), 9,852 (Cochrane Library) and 2,205 (EMBASE) articles. Thirteen articles were identified through other sources (reference lists). After initial screening and subsequent selection, a pool of 105 potentially relevant RCTs was identified. The subsequent eligibility process (Figure 1) excluded 91 articles. Among these, in six trials dynamic predictors of fluid responsiveness were used for GDFT in surgical patients, but the following reasons precluded their inclusion: studies comparing two GDFTdyn approaches [21-24], or studies comparing GDFTdyn to GDFT based on flow parameters [25], or studies using GDFTdyn but without any comparison group [26]. Two meeting abstracts fulfilled eligibility criteria [27,28], but were excluded because of not providing enough information in regard of methodology and patients outcomes and never making it to full publication. Overall, 14 articles [10,11,29-40] with a total sample of 961 patients, were considered for the analysis.

### Study characteristics

All selected articles were RCTs evaluating the effects of GDFTdyn on post-operative complications or length of stay. All studies were published between 2007 and 2013. All RCTs but two [30,33] were single-center trials. Eleven studies included major abdominal procedures, two cardiac surgeries and one a thoracic procedure. Eight studies were performed in Europe, three in China and one in each of the following countries: the United States, India and Brazil (Table 1).

**Table 1:** **Characteristics of included GDFTdyn studies**

**Study**	**Type of surgery**	**Risk**	**Number of participants** ^*^	**Timing**	**GDFTdyn end point**	**Other optimization goals**	**Type of intervention**	**Mortality reported**	**Morbidity reported**
Benes, 2010 [38] Europe	Major abdominal	High	120 (60/60)	Intra	SVV <10%	CI >2.5 l/min/m^2^, MAP >65 mmHg, CVP <15 mmHg,	Fluids, inotropes	Y	CV, GI, infectious, renal, respiratory
Buettner, 2008 [11] Europe	Major abdominal	Moderate	80 (40/40)	Intra	SPV <10%	N/A	Fluids	Y	-
Forget, 2010 [37] Europe	Abdominal	Moderate	82 (41/41)	Intra	PVI <10%	MAP >65 mmHg	Fluids	Y	CV, GI, infectious, renal
Goepfert, 2013 [36] Europe	Elective cardiac	High	100 (50/50)	Intra, post	SVV <10%	CI >2.0 l/min/m^2^, MAP >65 mmHg, HR 50-100 bpm, EVLWI ≤12 ml/kg	Fluids, inotropes	N	CV, infectious, respiratory, renal
Harten, 2008 [35] Europe	Emergency abdominal	High	29 (15/14)	Intra	PPV <10%	N/A	Fluids	Y	CV, GI, infectious, renal, respiratory
Kapoor, 2008 [39] India	Cardiac	High	27 (14/13)	Intra, post	SVV <10%	CI >2.5 mL/min/m^2^, CVP >6 mmHg, ScvO_2_ > 70%, SVI >30 ml/m^2^, SVRI >1,500 dynes.s/cm^5^/m^2^, DO_2_I >450 ml/min/m^2^	Fluids, inotropes	Y	GI, CV, renal, respiratory
Lopes, 2007 [10] Brazil	Major abdominal	High	33 (16/17)	Intra	PPV <10%	N/A	Fluids	Y	CV, GI, infectious, renal, respiratory
Mayer, 2010 [40] Europe	Major abdominal	High	60 (30/30)	Intra	SVV <12%	CI >2.5 mL/min/m^2^, MAP >65 mmHg, SVI >35 ml/m^2^	Fluids, inotropes	Y	Renal, respiratory
Ramsingh, 2013 [34] USA	Major abdominal	High	38 (20/18)	Intra	SVV <13%	N/A	Fluids	N	-
Salzwedel, 2013 [30] Europe	Elective abdominal	Moderate	160 (81/79)	Intra	PPV <10%	CI >2.5 mL/min/m^2^, MAP >65 mmHg	Fluids, inotropes	N	CV, GI, infectious, renal, respiratory
Sheeren, 2013 [33] Europe	Major abdominal	High	52 (26/26)	Intra	SVV <10%	SV increase >10%	Fluids	Y	CV, GI, infectious, renal, respiratory
Zhang Ju, 2012 [32] China	Major abdominal	Low	60 (20/40)	Intra	PPV <11%	N/A	Fluids	N	CV, GI, infectious, renal, respiratory
Zhang Ji, 2013 [31] China	Thoracic	Moderate	60 (30/30)	Intra	SVV <9%	CI >2.5 mL/min/m^2^, MAP >65 mmHg	Fluids, inotropes	N	GI, infectious, renal, Respiratory
Zheng, 2013 [29] China	Elective abdominal	Moderate	60 (30/30)	Intra, post	SVV <12%	CI >2.5 mL/min/m^2^, MAP >65 mmHg, SVI >35 ml/m^2^	Fluids, inotropes	N	CV, GI

^*^Number of participants displayed as overall (control/intervention); complications. GDFTdyn: goal-directed fluid therapy based on dynamic parameters; SVV: stroke volume variation; CV: cardiovascular; GI: abdominal (gastrointestinal/liver); MAP: mean arterial pressure; CVP: central venous pressure; SPV: systolic pressure variation; N/A, not available; PVI: pleth variability index; CI: cardiac index; HR, heart rate; EVLWI: extravascular lung water index; PPV: pulse pressure variation; SVI: stroke volume index; SVRI: systemic vascular resistance index; ScvO_2_: central venous oxygen saturation; DO_2_I: oxygen delivery index.

Missing or uncertain information was gathered by direct communication with the authors (see Acknowledgements section). Characteristics concerning the 14 RCTs analyzed are summarized in Table 1. Dynamic parameters used to guide fluid therapy were SVV in eight studies, PPV in four studies, SPV in one study and PVI in one study. SVV was measured by the FloTrac/Vigileo system (Edwards Lifesciences, Irvine, CA, USA) in seven studies and by the PiCCO system (Pulsion Medical Systems SE, Munich, Germany) in one study, PPV was measured by the bedside monitor in three studies and the ProAQT/Pulsioflex system (Pulsion Medical Systems SE) in one study, SPV was measured by the bedside monitor and PVI by the Radical 7 pulse oximeter (Masimo Corp, Irvine, CA, USA). The methodological evaluation, according to the SIGN score, showed that 10 out of 14 studies were considered as having low risk of bias (either ‘++’ or ‘+’ in Table 2).

**Table 2:** **Risk of bias assessed using the SIGN score**

**Study**	**SIGN score**	**SIGN comment**
Benes, 2010 [38] Europe	++	-
Buettner, 2008 [11] Europe	-	Blinding and concealment not clear
Forget, 2010 [37] Europe	++	-
Goepfert, 2013 [36] Europe	++	-
Harten, 2008 [35] Europe	-	Blinding not clear, outcomes not defined
Kapoor, 2008 [39] India	-	Randomization and blinding not clear, outcomes not defined
Lopes, 2007 [10] Brazil	++	-
Mayer, 2010 [40] Europe	+	Randomization not clear
Ramsingh, 2013 [34] USA	+	Intention-to-treat analysis not performed.
Salzwedel, 2013 [30] Europe	++	Multicentric trial
Sheeren, 2013 [33] Europe	+	Intention-to-treat analysis not performed, multicentric trial
Zhang Ju, 2012 [32] China	+	Intention-to-treat analysis not performed
Zhang Ji, 2013 [31] China	-	Randomization and blinding not clear, outcomes not defined
Zheng, 2013 [29] China	++	-

SIGN: The Scottish Intercollegiate Guidelines Network.

### Outcome measures

**Figure 2:**
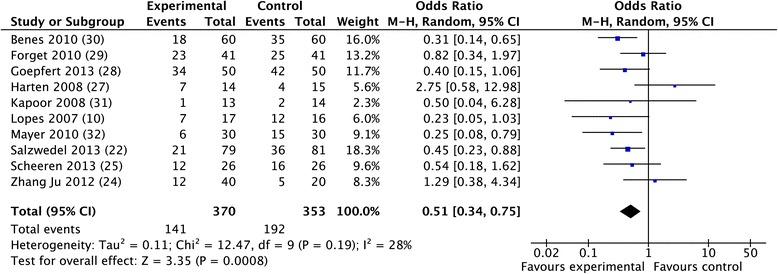
**Forest plot for post-surgical morbidity.**

**Figure 3:**
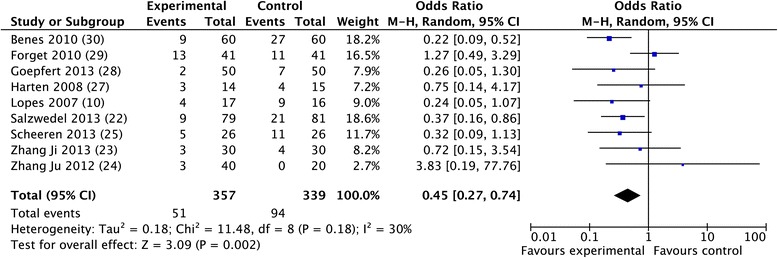
**Forest plot for infectious complications.**

**Figure 4:**
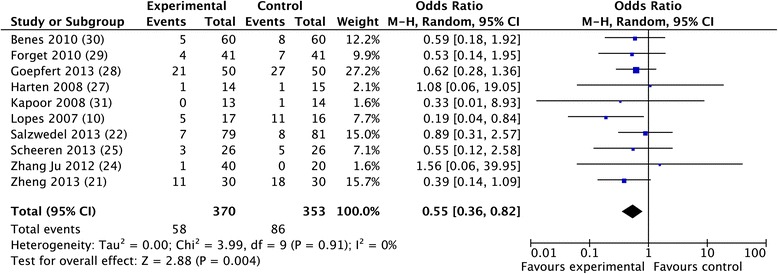
**Forest plot for cardiovascular complications.**

**Figure 5:**
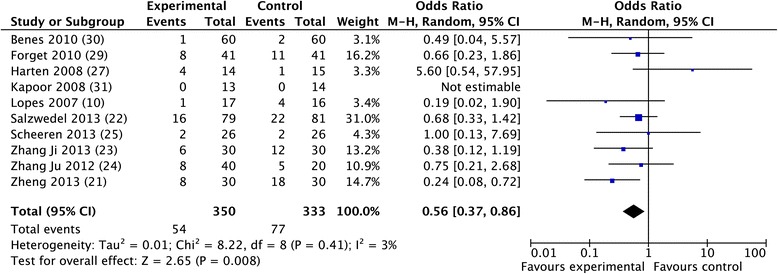
**Forest plot for abdominal complications.**

The overall morbidity rate was obtained from 10 studies [10,30,32,33,35-40] and a significant reduction was observed in favor of GDFTdyn (OR 0.51; CI 0.34 to 0.75; *P* <0.001; I^2^ = 28%) (Figure 2). A significant reduction in infectious (OR 0.45; CI 0.27 to 0.74; *P* = 0.002; I^2^ = 30%), cardiovascular (OR 0.55; CI 0.36 to 0.82; *P* = 0.004; I^2^ = 0%) and abdominal complications (OR 0.56; CI 0.37 to 0.86; *P* = 0.008; I^2^ = 3%) was observed in favor of GDFTdyn (Figures 3, 4 and 5). A non-significant trend towards a reduction in respiratory complications was observed (0.60; CI 0.33 to 1.09; *P* = 0.09; I^2^ = 0%). Renal complications were not significantly reduced by GDFTdyn (0.57; CI 0.24 to 1.35; *P* = 0.20; I^2^ = 40%).

**Figure 6:**
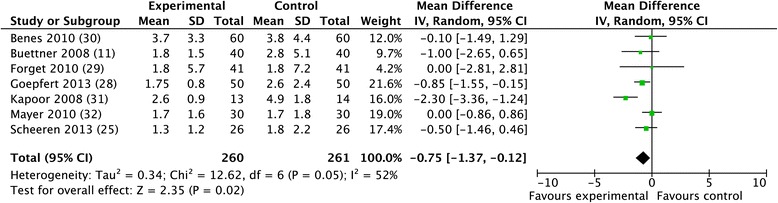
**Forest plot for ICU length of stay.**

A significant reduction in ICU length of stay was also observed (WMD −0.75 days; CI −1.37 to −0.12; *P* = 0.02; I^2^ = 52%) (Figure 6), whereas hospital length of stay did not significantly decrease (WMD −1.33 days; CI −2.90 to 0.23; *P* = 0.10; I^2^ = 78%).

## Discussion

Our meta-analysis shows that GDFTdyn decreases post-surgical morbidity, the rate of infectious, cardiac and abdominal complications, as well as ICU length of stay.

Many post-surgical complications are related, at least in part, to insufficient or excessive fluid administration during the peri-operative period [41]. A U-shaped relationship is classically described between the amount of fluid administered peri-operatively and the morbidity rate [41]. It has been suggested that giving fluid until patients’ heart has reached the plateau of the Frank-Starling relationship may be the most efficient way to prevent both hypovolemia and fluid overload. In clinical practice, this approach consists of giving fluid until flow parameters (stroke volume or cardiac output) reach a plateau value (to prevent hypovolemia), then to stop giving any additional fluid volume (to prevent fluid overload).

Dynamic predictors of fluid responsiveness are not markers of blood volume, nor markers of cardiac preload, but markers of the position on the Frank-Starling curve [2]. In this regard, they have been proposed to identify when the plateau of the Frank-Starling relationship is reached without the need to give fluid and to monitor flow parameters [42]. Several randomized controlled trials and meta-analyses have demonstrated the superiority of GDFT based on flow parameters over standard fluid management to decrease renal, gastrointestinal, respiratory and infectious complications, as well as the overall morbidity rate [43-50]. From a physiological standpoint, maximizing stroke volume or minimizing dynamic parameters with fluid are equivalent [42]. Therefore, one can expect similar benefits when using dynamic parameters than when using flow parameters to guide fluid therapy. This is what our meta-analysis does confirm: clinical benefits of GDFTdyn are comparable to those reported with GDFT based on flow parameters [46,51].

Fuelled by the growing number of clinical studies and meta-analyses demonstrating the value of GDFT, official recommendations have been published [52]. However, despite these recommendations, adoption of GDFT remains poor [53]. A recent survey showed that a minority of anaesthetists use GDFT in patients undergoing high-risk surgery, whereas they believe they should [9].

For GDFT, the use of dynamic parameters has several potential advantages over the use of flow parameters. First, it has the advantage of being simple, whereas the use of flow parameters requires interventions and calculations. For instance, the stroke volume fluid optimization strategy, currently recommended both in the UK and in France [54,55], requires the assessment and quantification of the percentage change in stroke volume during a standardized fluid challenge. Oxygen delivery optimization strategies require intermittent calculations of the oxygen delivery index based on the simultaneous measurement of cardiac output, hemoglobin and arterial oxygen saturation by different devices. As a result, fluid strategies based on flow parameters are often perceived as complex and time-consuming by caregivers. In contrast, using dynamic parameters does not require any intervention to know if the patient is a fluid responder or not (a high SPV, PPV, SVV or PVI value suggests that the patient is fluid responsive), nor any calculations (delta change in stroke volume, oxygen delivery). Caregivers simply have to monitor dynamic parameters and ensure the value remains below a predefined target value (usually around 10 to 12%). Simplicity may be a key element to expand the clinical adoption of GDFT. Second, using dynamic parameters may be considered as a cost-saving approach. Indeed, although SVV measurement requires the use of a cardiac output monitor, the estimation of PPV is possible from any bedside monitor displaying an arterial pressure curve. Of note, the mere eyeballing of the respiratory swings in arterial pressure may be misleading such that a real quantification of this phenomenon is required [2]. A minority of monitors currently in use in operating rooms have the ability to automatically calculate PPV. Freezing the arterial pressure tracing, and measuring systolic and diastolic pressures beat by beat to identify the maximum and minimum pulse pressure values over a single respiratory cycle is a real but time-consuming alternative to the automatic calculation of PPV.

The use of dynamic parameters has also disadvantages. The main disadvantage is the fact that they cannot be used in many patients because of limitations, which have been described in detail elsewhere [2], and do include small tidal volume (<8 ml/kg), open chest, sustained cardiac arrhythmia and abdominal hypertension (for example laparoscopy) [56]. A study [57] looking at more than 12,000 non-cardiac surgical patients concluded that, given their limitations, dynamic parameters could have been used to guide fluid therapy in only 39% of the cases, the most frequent limitation being the use of a small tidal volume for mechanical ventilation (one-third of the cases in this specific study). To decrease the risk of ventilation-induced lung injury, clinicians have lowered tidal volumes, not only in patients with respiratory failure, but also in patients with healthy lungs undergoing surgery. A recent study by Futier *et al*. [58] shows that using tidal volumes of 6 to 8 ml/kg during abdominal surgery is associated with a better post-operative outcome than when using a tidal volume of 10 to 12 ml/kg. If such low tidal volumes (6 to 8 ml/kg) were to be adopted widely to ventilate patients in operating theatres, this would significantly decrease the applicability of dynamic parameters for GDFT. However, this study [58] does not disqualify the use of intermediate tidal volumes (8 to 10 ml/kg) that would be compatible with the use of dynamic parameters for GDFT.

The main limitation of our study is the heterogeneity of the randomized controlled trials we analyzed. First, we observed a statistical (the Q and I^2^ tests) heterogeneity and inconsistency among studies investigating the effects of GDFTdyn on ICU and hospital length of stay, as well as on renal function. Previous meta-analyses have shown a significant reduction in renal insufficiency with GDFT [43]. A benefit has also been reported for hospital length of stay, with a reduction ranging between one and two days [51,59]. In contrast, previous meta-analyses failed to demonstrate a decrease in ICU length of stay. Therefore, the effect or lack of effect of GDFTdyn on ICU and hospital length of stay, as well as on renal function, deserves further investigation before drawing any definitive conclusion. Beside the heterogeneity detected by statistical tests, one must acknowledge that the randomized controlled trials were also different with regard to the definition of post-surgical complications. Unfortunately, there is no universal definition for post-surgical complications such as bacterial pneumonia (protected or non-protected bacteriological samples) or acute renal insufficiency (oliguria or increase in creatinine level), and this certainly contributes to the heterogeneity of the studies we analyzed. If fluid management was always based on the monitoring of dynamic parameters, different trigger values, ranging from 9 to 13% (Table 1), were used to give fluid. In addition, some hemodynamic protocols included additional static parameters such as blood pressure and cardiac output (Table 1). The heterogeneity among treatment protocols and definition of complications is a common feature of previous GDFT meta-analyses [43-50], but it is important to bear in mind that it may have influenced our findings.

## Conclusion

Our meta-analysis suggests that GDFTdyn decreases post-surgical morbidity, infectious, cardiac and abdominal complications, as well as ICU length of stay. Pending limitations to their use being understood and respected, dynamic parameters may represent an alternative to flow parameters to guide peri-operative fluid therapy. Because of the heterogeneity of studies analyzed, large prospective clinical trials would be useful to confirm our findings.

## Key messages

- We analyzed 14 randomized controlled trials investigating the value of goal-directed fluid therapy (GDFT) based on dynamic parameters (GDFTdyn) to improve post-surgical outcome.
- Our meta-analysis showed that GDFTdyn is associated with a significant decrease in post-surgical morbidity, defined as the proportion of patients developing one or more complication.
- This decrease in post-surgical morbidity was related to a significant reduction in infectious, cardiovascular, and abdominal complications and was associated with decrease in ICU length of stay.
- GDFTdyn may be an interesting alternative to classical GDFT strategies, which are based on flow parameters such as cardiac output or oxygen delivery.

## Additional files

Additional file 1:
**PRISMA checklist.**
Additional file 2:
**Search strategies.**

## Abbreviations

CI: confidence interval
GDFT: goal-directed fluid therapy
GDFTdyn: goal-directed fluid therapy based on dynamic parameters
OR: odds ratio
PPV: pulse pressure variation
PVI: pleth variability index
RCTs: randomized controlled trials
SIGN: The Scottish Intercollegiate Guidelines Network
SPV: systolic pressure variation
SVV: stroke volume variation
WMD: weight mean difference
